# The Loudness of Suctioning in the Ear Canal

**DOI:** 10.7759/cureus.14208

**Published:** 2021-03-31

**Authors:** Colin Byrd, Eytan Keidar, Olga Santiago, Carl Shermetaro

**Affiliations:** 1 Otolaryngology - Head and Neck Surgery, McLaren Oakland Hospital, Pontiac, USA; 2 Graduate Medical Education, McLaren Oakland Hospital, Pontiac, USA

**Keywords:** noise induced hearing loss, sensorinerual hearing loss, aural toilet, ear suctioning

## Abstract

Aim

To determine the loudness of suctioning in the ear canal with different-sized suctions and various mediums. Aural microsuctioning is commonly used in the otolaryngologist’s office setting for cerumen removal and aural toilet. We hypothesize that the intensity of the sound would increase with increasing viscosity of the medium and increasing suction diameter.

Methods

The intensity of the sound generated was measured while suctioning air, water, and yogurt on cadaveric temporal bones with size 7 and 5 Frazier suctions. This was performed with one measurer and one operator. Under otomicroscopy, the operator would suction the ear canal and the measurer would record the intensity of the sound with a sound decibel meter placed at the lateral and posterior external auditory canal. Data was collected with two separate operators and measurers to aid with inter-rater reliability.

Results

There was a total of 240 repeated observations (10 cadavers, 3 mediums, 2 suction devices; 2 investigators). The range of the maximum peak intensity ranged from 63.0 dB to 100.0 dB. The lowest peak intensity of decibels was recorded in air with the size 5 Frazier suction; and the highest measured was with the size 5 Frazier suction in yogurt. Statistically significant differences were found only in the measurements in air.

Conclusion

Our investigation found that increasing peak sound intensities were generated by increasing the viscosity of the fluid medium that was being suctioned. However, the smaller sized diameter suction actually generated louder sound intensities than the larger diameter suction with higher viscosity fluid media, but this was not statistically significant.

## Introduction

A large percentage of patients who present to the otolaryngologist require routine ear cleaning for multiple reasons, including cerumen impaction, otorrhea secondary to otitis externa, otitis media, and various other etiologies. Otolaryngologists routinely clean out patients’ ears using strategies that include otomicroscopy with a curette, alligator forceps, and suction. Otomicroscopy is generally preferable as it allows for concomitant examination, while irrigation may be uncomfortable and result in dizziness.

Newer ear cleaning strategies include soaking the canal with hydrogen peroxide or saline solution, which can aid with removal [1]. A Cochrane review actually revealed that any cerumonolytic is better than none but no one cerumonolytic appears to be superior [1]. Suctioning out fluid from the ear canal can be loud and uncomfortable, and the larger the diameter of the Frazier-type suction, the more powerful and louder the suction. We routinely use a size 7 Frazier suction in the ear canal, but as we move closer to the tympanic membrane, we tend to transition to smaller sized suctions such as a size 5 or 3. The viscosity of the fluid medium in the ear canal can also play a role in the perceived sound intensity of suctioning in the ear canal. The speed of sound travels fastest through solids because molecules are more densely packed together.

Sound travels over 1400 meters per second in liquid, which is over four times faster than in air [2]. There is limited clinical data on the topic of this study. For example, Nelson and colleagues found in their prospective study on 21 patients that a peak intensity of 111 decibels (dB) was recorded with suctioning water from the ear canal, there was no reported hearing loss or shift in hearing thresholds [3]. Other studies performed with mannequins, anatomic silicon ear models, or adult cadaveric human temporal bones have reported suction sound levels from 68.3 dB to 129 dB [3,4,5]. Some factors that have affected the range in dB reported in previous studies are the medium used (e.g., air, water, other fluids), the Frazier size (e.g., #5. #7, and #9), and the recording technique. For example, Ross suctioned with size 3, 5, and 7 suctions at various positions in the ear canal on a mannequin and found the max intensity to be 114.4 dB [4]. In a similar study, Snelling and colleagues recorded two episodes of greater than 120 dB on a routine aural toilet with microsuction and a mean of 102.5 dB by just inserting the suction into the ear canal [5]. Most recently, Young and colleagues reported sound levels from 68.3 dB to 97 dB with size 3, 5, and 7 Fraziers at various positions in the ear canal in wet and dry conditions [6]. In their study, they found statistically significant differences based on the diameter of the suction, where larger suctions generated louder dB [6].

The purpose of this study was to determine the sound intensity of suctioning in the ear canal with different sized Frazier suctions on various fluid mediums (i.e., air, water, yogurt). It is our hypothesis that suctioning medium of higher viscosity with larger suction sizes will result in increased loudness.

The study was not subject to direct IRB oversight because it was deemed non-human research.

## Materials and methods

Medium

The loudness of suctioning in the ear canal was measured with air, water, and yogurt to replicate infection, cerumen, and other ear canal debris that are normally seen in patients. To avoid altering the data, we performed the measurements first in air, second in water, and third in yogurt. The yogurt was used to replace purulent otorrhea in the setting of infection or thick, soft cerumen. The viscosity of yogurt along with multiple other dairy products has been measured previously. For example, the absolute viscosity of yogurt has been reported as 152 at 40°C [7]. In this study, we used Stonyfield® Organic Kids yogurt (Stonyfield Farm, Inc., Londonderry, NH, USA). It was injected with an 18-gauge needle on a syringe filling the entire ear canal beginning near the tympanic membrane. Since the anatomy and size of each ear canal varies, instead of using a fixed amount of fluid, the ear canal was filled until the medium (i.e., water or yogurt) began to spill out.

Cadavers

This study used a sample of 10 cadaveric temporal bones that were available at our laboratory at McLaren Oakland Hospital. Each bone was labeled from 1 to 10 (e.g., #1, #2, #3, etc.). All cadaveric temporal bones were not tampered with prior to this investigation and they had their mastoids intact, except for temporal bone #5, which had an apparent mastoidectomy during their lifetime. There was a pinpoint tympanic membrane perforation on temporal bone #1.

Measurement of decibels

The loudness of suctioning in the ear canal was measured on the various media using a size 7 and 5 Frazier-type suction in each cadaveric temporal bone and was recorded by two separate recorders. Our methods followed those of Snelling et al. [5], however, we used cadaveric temporal bones and their study used human subjects.

The loudness was measured in decibels with a mini digital sound decibel level meter less than 6" in length. It has the capacity to measure from 40 dB to 130 dB, which is in the range of the peak intensity reported in previous studies. The accuracy of the meter reported is +/- 3.5 dB @1 kHz, 94 dB under reference conditions. The mini sound decibel meter was placed consistently at the lateral and posterior edge of the external auditory canal (EAC) over the mastoid. The same size 7 and 5 suction devices were used for the entire experiment.

A standard suction wall unit was used on a setting of 100 mmHg and attached to 12 ft of nonconductive suction tubing. The peak intensity was recorded as the medium was suctioned from the ear canal. All measurements were taken with one operator managing the suction on a cadaveric temporal bone under otomicroscopic visualization and a recorder holding the mini digital sound decibel meter at the edge of the EAC. Two separate consecutive peak sound measurements were recorded for each suction size in each individual medium. To develop measurement skills and decrease the potential confounding of a learning curve, the investigator practiced with each medium before performing the official measurements.

## Results

Before any data analysis, we tested for normality distribution of decibels by type of medium and possible outliers. The results of the loudness were reported by medium and suction size. Decibels are measured as a continuous variable, the results are presented as frequencies, mean, median, and range. To perform the statistical analyses, we transformed the decibels measurements by first dividing the decibel data point by 10; second, calculating the anti-log of that value. Since the decibels by medium did not have a normal distribution, we performed tests on the equality of standard deviations (variances), which guided us to perform either a two-sample t-test for unequal variances or for equal variances. Thereafter, we reversed the mathematical transformation to convert the statistical estimates (i.e., means and standard deviations) back to decibel levels. 

There was a total of 240 repeated observations (i.e., 10 cadavers, 3 mediums, 2 suction devices; 2 investigators). We did not identify any outliers by medium. There were no statistically significant (p > 0.05) differences between the two decibel’s measurements by medium. Therefore, we decided to use the first of the two consecutive measurements to calculate the means and to test for mean differences by suction size in each medium. In this analysis, we stressed the precision of the study estimates with a focus on 95% confidence intervals and p-values will be presented as an aid to interpretation. All analyses were performed with STATA 15.1 (StataCorp LLC, College Station, TX, USA). 

The range of the maximum peak intensity ranged from 63.0 dB to 100.0 dB. The lowest peak intensity of decibels was recorded in air with the size 5 suction; and the highest with yogurt also with the size 5 suction device. When testing if there were statistically significant differences in decibels by suctions sizes in each medium, we only found differences in the measurements performed in air (Table 1). 

**Table 1:** Sound decibel levels by medium and suction size a- Two-sample t-test with unequal variances b- Two-sample t-test with equal variances LL = lower limit, UL = upper limit, CI = confidence interval, SD = standard deviation

		Range				95% CI		
Medium	Suction size	Minimum	Maximum	Mean	SD	LL	UL	p-value
Air	All (n=40)	63.01	79.29	70.57	71.93	68.04	72.15	
	Suction #5	63.01	72.55	76.99	66.07	64.92	68.38	0.01^a^
	Suction #7	63.80	79.29	72.48	73.01	69.22	74.33	
Water	All (n=40)	70.57	95.56	87.19	88.39	84.84	88.72	0.706^b^
	Suction #5	74.62	95.56	87.53	89.14	82.60	89.78	
	Suction #7	81.46	93.42	86.84	87.32	83.62	88.66	
Yogurt	All (n=40)	83.34	100.00	94.18	93.28	92.88	95.19	0.566^a^
	Suction #5	83.22	100.00	94.50	94.31	91.90	96.11	
	Suction #7	87.71	97.48	93.84	91.43	92.48	94.87	

## Discussion

This study is unique to the literature because it determined that not only the size of the suction but also the viscosity of the fluid medium affects the intensity of the sound perceived by the patient undergoing aural microsuction. Although the suctioning was performed on cadaveric temporal bones, the data can be extrapolated and applied to human subjects in the clinical setting. As such, clinicians must be aware of the potential risks of even minor procedures like suctioning in the ear canal which may result in temporary threshold shifts. These events are essentially a result of reversible damage to the outer hair cells of the inner ear that may be accompanied by tinnitus, muffled hearing, and loudness recruitment [8]. Recovery can range from minutes to days, with multiple exposures or a threshold shift greater than 40 dB, recovery may be delayed or incomplete resulting in a permanent threshold shift [8]. These temporary threshold shifts may occur with intensities greater than 70 dB [8]. The exact relationship between the temporary threshold shift and permanent threshold shift stages of hearing loss caused by noise exposure is unknown [8].

Hearing loss is the third most common chronic condition in the U.S., even more prevalent than diabetes or cancer [9]. Noise-induced hearing loss is the second most common cause of hearing loss behind age-related hearing loss [10]. Noise exposures greater than or equal to 100 dB for a duration of 1 hour or greater than 85 dB for 8 hours per day can result in noise-induced sensorineural hearing loss [8]. The degree of damage to the inner ear is proportional to the intensity and duration of sound exposure [8]. The risk of hearing loss increases with the intensity of the sound, duration of exposure, number of exposures, and the susceptibility of the patient [8]. Noise-induced hearing loss is an insidious process and results in irreversible damage to the outer hair cells of the inner ear [8]. This damage is seen objectively on an audiogram with a noise notch seen at 3,000, 4,000, or 6,000 Hz with recovery at 8,000 Hz [8].

The relationship between occupational noise exposure and hearing loss has become better understood, and as a result, more emphasis has been placed on protective measures. Occupational Standards and Health Administration (OSHA) is a federal agency that regulates safe working environments. OSHA standards require that workplace noise exposure greater than 85 dB require protective measures and conservation programs to prevent noise-induced hearing loss [3]. The World Health Organization recommends no sounds above 120 dB are permissible. Additionally, the US American Conference of Governmental Industrial Hygienists and the National Institute of Occupational Safety and Health specify 103 dB as the maximum recommended unprotected noise exposure [4].

This study attempted to account for the variation in sound intensity with suctioning in the ear canal as a result of the change in the viscosity of the fluid and the size of the suction device. The intensity of the sound was louder with a smaller diameter suction in water and yogurt but this was not statistically significant. Suctioning yogurt with a size 5 suction frequently resulted in sound intensities greater than 94 dB and a maximum intensity of 100 dB. Our data differed from the data of Ross, who found that suctioning with the larger diameter or number 7 suction resulted in the loudest recorded sound and they recorded peaks up to 140 dB, which is much higher than our max of 100 dB. The exact viscosity of Stonyfield® Organic Kids yogurt is not known, however, various studies have looked into the physical properties of yogurt in the food industry. For example, Yu’s study found the kinematic viscosity of yogurts with whole milk is about 2 centistokes at 22°C [11]. The kinematic viscosity of water at 20°C is 1 centistoke [11].

Ear canal volume also appears to contribute to sound intensity. Anecdotally, in this experiment, the smaller ear canals appeared to have louder sound intensities with suctioning. Ear canal volumes were not objectively measured in this study. Also, in Nelson’s study, there did appear to be a negative correlation between ear canal volume and sound intensity with suctioning.

The use of cadaveric temporal bones is a limiting factor in this study, although, they did have soft tissue still present, but the auricle was removed. Also, we were limited to 10 temporal bones at our institution. This study did not account directly for ear canal volume, middle ear, or mastoid aeration, which all likely contribute to the resonance of sound when suctioning. Further investigation especially in live humans is needed.

## Conclusions

In this study, as the viscosity of the fluid medium increased, and as the size of the suction decreased, so did the loudness recorded. Suctioning yogurt with a size 5 suction frequently resulted in sound intensities greater than 94 dB. This is of particular interest in stenotic ear canals, pediatric patients, and those with otitis externa where their ear canal is narrowed and potentially swollen. The narrowed ear canal with purulent fluid medium results in loud sound intensities with aural toilet. This study did not account directly for ear canal volume, middle ear, or mastoid aeration, which all likely contribute to the resonance of sound when suctioning. The utilization of smaller suctions like size 5 appears to contribute even more to the problem.

Noise-induced hearing loss is a significant contributor to adult-acquired sensorineural hearing loss. It is largely preventable and is an insidious process that occurs over many years.

Primary care physicians must be aware of the implications of noise-induced hearing loss, particularly in those that work in construction and manufacturing who are at the highest risk. Education, prevention with ear protection, and referral to an otolaryngologist early in the disease process are essential as early intervention with hearing aids has shown to be important for hearing preservation, quality of life, and prevention of cognitive decline and depression. Clinicians should be cautious even when performing routine procedures such as ear cleanings as this may be loud enough to result in temporary threshold shifts.

## References

[1] Burton MJ, Doree C (2018). WITHDRAWN: Ear drops for the removal of ear wax. Cochrane Database Syst Rev.

[2] Moebs W, Ling SJ, Sanny J (2016). Speed of sound. University Physics Volume 1.

[3] Nelson JJ, Giraud A, Walsh R, Mortelliti AJ (2011). Impact on hearing of routine ear suctioning at the tympanic membrane. Am J Otolaryngol.

[4] Ross JR (2009). Re: Noise levels generated within the external auditory canal during microsuction aural toilet. Clin Otolaryngol.

[5] Snelling JD, Smithard A, Waddell A (2009). Noise levels generated within the external auditory canal during microsuction aural toilet and the effect on hearing: a prospective controlled series. Clin Otolaryngol.

[6] Young A, Reeve NH, Yang A, Kahane J, Cross C, Albanese A, Ng M (2020). Sound levels with aural suctioning: Effects of suction size, canal moisture, and distance from the eardrum. Laryngoscope Investig Otolaryngol.

[7] Smith Smith, Michael Michael (2020). Approximate viscosities of some common liquids. https://www.michael-smith-engineers.co.uk/resources/useful-info/approximate-viscosities-of-common-liquids-by-type.

[8] Adunka OF, Buchman CA (2011). Otology, Neurotology, and Lateral Skull Base Surgery: An Illustrated Handbook. Otology, neurotology, and lateral skull Base Surgery.

[9] Masterson EA, Bushnell PT, Themann CL, Morata TC (2016). Hearing impairment among noise-exposed workers - United States, 2003-2012. MMWR Morb Mortal Wkly Rep.

[10] Flint P, Haughey B, Lund V (2015). Cummings Otolaryngology, Head and Neck Surgery. Cummings Otolaryngology, Head and Neck Surgery.

[11] Yu HY, Wang L, McCarthy KL (2016). Characterization of yogurts made with milk solids nonfat by rheological behavior and nuclear magnetic resonance spectroscopy. J Food Drug Anal.

